# First Experimental Evidence for the Presence of Potentially Toxic *Vibrio cholerae* in Snails, and Virulence, Cross-Resistance and Genetic Diversity of the Bacterium in 36 Species of Aquatic Food Animals

**DOI:** 10.3390/antibiotics10040412

**Published:** 2021-04-09

**Authors:** Dailing Chen, Xiangyi Li, Ling Ni, Dingxiang Xu, Yingwei Xu, Yong Ding, Lu Xie, Lanming Chen

**Affiliations:** 1 Key Laboratory of Quality and Safety Risk Assessment for Aquatic Products on Storage and Preservation (Shanghai), Ministry of Agriculture and Rural Affairs of the People’s Republic of China, College of Food Science and Technology, Shanghai Ocean University, Shanghai 201306, China; 180310748@st.shou.edu.cn (D.C.); Lixy376@mail2.sysu.edu.cn (X.L.); lni@shou.edu.cn (L.N.); 190300759@st.shou.edu.cn (D.X.); 190300760@st.shou.edu.cn (Y.X.); yding@shou.edu.cn (Y.D.); 2 Shanghai Center for Bioinformation Technology, Shanghai 201203, China

**Keywords:** *Vibrio cholerae*, virulence, antibiotic resistance, heavy metal tolerance, genetic diversity, aquatic food animals

## Abstract

*Vibrio cholerae* is the most common waterborne pathogen that can cause pandemic cholera in humans. Continuous monitoring of *V. cholerae* contamination in aquatic products is crucial for assuring food safety. In this study, we determined the virulence, cross-resistance between antibiotics and heavy metals, and genetic diversity of *V. cholerae* isolates from 36 species of aquatic food animals, nearly two-thirds of which have not been previously detected. None of the *V. cholerae* isolates (n = 203) harbored the cholera toxin genes *ctxAB* (0.0%). However, isolates carrying virulence genes *tcpA* (0.98%), *ace* (0.5%), and *zot* (0.5%) were discovered, which originated from the snail *Cipangopaludina chinensis*. High occurrences were observed for virulence-associated genes, including *hapA* (73.4%), *rtxCABD* (68.0–41.9%), *tlh* (54.2%), and *hlyA* (37.9%). Resistance to moxfloxacin (74.9%) was most predominant resistance among the isolates, followed by ampicillin (59.1%) and rifampicin (32.5%). Approximately 58.6% of the isolates displayed multidrug resistant phenotypes. Meanwhile, high percentages of the isolates tolerated the heavy metals Hg^2+^ (67.0%), Pb^2+^ (57.6%), and Zn^2+^ (57.6%). Distinct virulence and cross-resistance profiles were discovered among the *V. cholerae* isolates in 13 species of aquatic food animals. The ERIC-PCR-based genome fingerprinting of the 203 *V. cholerae* isolates revealed 170 ERIC-genotypes, which demonstrated considerable genomic variation among the isolates. Overall, the results of this study provide useful data to fill gaps for policy and research related to the risk assessment of *V. cholerae* contamination in aquatic products.

## 1. Introduction

*Vibrio cholerae* is the causative agent of cholera, a life-threatening diarrheal disease that is typically transmitted via contaminated water and person-to-person contact [1]. The bacterium has caused seven cholera pandemics since 1817 [2]. The seventh pandemic, which erupted in 1961, has lasted for over 50 years [3]. It is estimated that around 2.86 million cholera cases occur globally every year, claiming 120,000 lives. About 1.3 billion people are currently at risk of infection from cholera [4]. According to World Health Organization (WHO) reports, cholera has remained endemic in developing countries in recent years (https://www.who.int/, accessed on 26 July 2020), where sanitation conditions are poor and drinking water unsafe. 

*V. cholerae* is found growing in aquatic environments worldwide [2]. Epidemic *V. cholerae* strains produce cholera toxin (CT) and toxin-coregulated pili (TCP) [5]. The CT-encoding genes (*ctxAB*) are found within a ~7 kb filamentous bacteriophage (designated CTXΦ). CTXΦ uses TCP as its receptor and infected *V. cholerae* cells within host gastrointestinal tracts. The TCP-encoding genes are clustered within a ~41 kb region known as vibrio pathogenicity island (VPI) [5]. Virulence-associated genes that contribute to its pathogenicity include those encoding zonula occludens toxin (*zot*), accessory cholera enterotoxin (*ace*), RTX toxin (*rtxCABD*), El Tor hemolysin (*hlyA*), hemagglutinin protease (*hapA*), thermolabile hemolysin (*tlh*), mannose-sensitive hemagglutination pili (*mshA*), and putative type IV pilus (*pilA*) [6]. *V. cholerae* is frequently isolated from aquatic products including fish, shellfish, and crustaceans [6,7,8,9]. China is the largest producer, consumer, and exporter of aquatic products in the world, accounting for about 60% (64,803,600 tons) of the global total in 2019 (National Bureau of Statistics, http://www.stats.gov.cn/, accessed on 17 February 2021). Therefore, continuous monitoring of *V. cholerae* contamination in aquatic products and identification of risk factors are imperative for assuring food safety. 

Antimicrobial agents can effectively prevent the spread of the infectious disease, however, the overuse and misuse of antimicrobial agents result in antibiotic resistant *V. cholerae* [10]. For example, Iramiot et al. investigated the antibiotic resistance of *V. cholerae* associated with the 2017 cholera outbreak in Kasese District, Uganda. They found that *V. cholerae* strains (n = 69) were highly resistant to commonly used antibiotics such as ampicillin (AMP, 100%) and trimethoprim/sulfamethoxazole (SXT, 50%) [11]. Aquatic environments are bulk *V. cholerae* reservoirs and might be an important source of resistant strains [12]. Numerous studies have reported that *V. cholerae* isolates recovered from aquatic environments showed multidrug resistant (MDR) phenotypes [6,12,13,14]. For example, Sulca et al. reported that two *V. cholerae* strains isolated from Lima, Peru, seawater were resistant to 12 antimicrobial drugs [14]. In our prior research, we examined 400 *V. cholerae* isolates recovered from four species of fish collected in 2017 in Shanghai, China, and found high incidence of resistance to streptomycin (STP) (65.3%), AMP (44.5%), and rifampicin (RIF) (24.0%) among the isolates [6]. 

Aquatic environments polluted with toxic heavy metals are also a challenging issue particularly in developing countries [15]. Due to their nondegradable nature, the high bioaccumulation of heavy metals through the food chain poses serious threats to human health and well-being [16]. It may also enhance the selection for antibiotic resistance and vice versa [6,17]. Residues of hazardous heavy metals have been found in waters, sediments and aquatic products sampled worldwide, particularly in developing countries [18,19,20]. For example, Zhang et al. analyzed the concentrations of eight heavy metals (As, Cd, Cr, Cu, Hg Ni, Pb, and Zn) in 54 surface sediment samples collected in Subei Shoal, Jiangsu Province, China in July 2017. Their data showed that Hg (0.06 ± 0.02 mg/kg sediment) and Cd (0.56 ± 0.77 mg/kg sediment) were the major pollution factors with moderate pollution levels [18]. In our earlier research, tolerance to Hg, Cd, Pb, Cu, and/or Zn was found in several *V. cholerae* strains isolated from the surface water of the Yangtze River Estuary in Shanghai, where the East China Sea is one of the major fishing grounds along China’s coast [21]. We hypothesized that there could be heavy metal resistant *V. cholerae* in aquatic products. Nevertheless, the available literature in this field is rare. Recently, we analyzed *V. cholerae* isolates recovered from 12 species of aquatic products sampled in Shanghai in 2017 and 2018, and found high incidence of tolerance of the isolates to Hg, Ni, Cd, Zn, and/or Pb [6,9]. 

In the present study, we conducted a survey on a larger scale to investigate virulence, cross-resistance between antibiotics and heavy metals, and genetic diversity of *V. cholerae* recovered from 36 species of aquatic food animals sampled in July and August of 2019 in Shanghai and Fuzhou, located in the southeastern littoral area of China. To our knowledge, *V. cholerae* has not been detected previously in 22 species of aquatic animals. The results in this study provide useful data to fill gaps in the risk assessment of aquatic products and to better understand the mechanism underlying cross-selection between antibiotics and heavy metals in the leading waterborne pathogen worldwide.

## 2. Results

### 2.1. Prevalence of V. cholerae in 36 Species of Aquatic Food Animals

A total of 1754 yellow single colonies were recovered from the 36 species of aquatic food animals sampled in this study. They were randomly selected from selective TCBS agar plates for further identification. Approximately 20.3% (356 of 1754 colonies) of the colonies tested positive for the *V. cholerae*-specific gene *lolB*, but negative in double-arginine hydrolase and esculin hydrolysis tests. The results were verified by DNA sequencing and analysis of the *lolB* and 16S rRNA genes. Among the 356 *V. cholerae* isolates, approximately 64.3%, 24.7%, 8.1%, and 2.8% originated from fish (229 of 696 colonies), snails (88 of 427 colonies), shellfish (29 of 618 colonies), and crab (10 of 10 colonies), respectively, while none from shrimp (0 of 3 colonies) samples.

Of the 36 species of aquatic food animals, *V. cholerae* was examined for the first time in 22 species, including seven species of fish (*Blotchy rock Cod, Ditrema temmincki Bleeker*, *Hemiculter leucisculus*, *Misgurnus anguillicaudatus*, *Monopterus albus*, *Nibea albiflora* and *Plectorhynchus cinctus*); nine species of shellfish (*Golden scallop*, *Mactra antiquata*, *Mactra veneriformis*, *Musculus senhousei*, *Mytilus edulis*, *Pseudocardium sachalinense*, *Saxidomus purpuratus*, *Scapharca subcrenata* and *Tegillarca granosa*); four species of snails (*Babylonia areolata, Babylonia lutosa, Cipangopaludina chinensis* and *Neptunea cumingi Crosse*); one species of shrimp (*Procambarus clarkii*); and one species of crab (*Eriocheir sinensis*) (Table S1). 

The results revealed that V. cholerae was present in only 13 species of aquatic animals, including six species of fish (Aristichthys nobilis, Carassius auratus, Lateolabrax japonicas, M. anguillicaudatus, Mylopharyngodon piceus, and Parabramis pekinensis), three species of shellfish (G. scallop, Ostrea gigas Thunberg, and Solen strictus), three species of snails (B. areolata, C. chinensis and N. cumingi Crosse), and the crab E. sinensis (Table S1). Among these, V. cholerae was isolated for the first time from 6 species: M. anguillicaudatus, G. scallop, B. areolata, C. chinensis, N. cumingi Crosse, and E. sinensis. Conversely, the bacterium was absent from the other 23 species of aquatic food animal samples examined in this study (Table S1). 

### 2.2. Virulence-Associated Gene Profiles of the V. cholerae Isolates

All the identified *V. cholerae* isolates recovered from the shellfish (n = 29) and crab (n = 10) samples were subjected to the further analysis, while some were randomly chosen from the fish (n = 95) and snails (n = 69). A total of 203 *V. cholerae* isolates were tested in the following experiments. None of the isolates harbored the cholera toxin genes *ctxAB*. However, the *tcpA* virulence gene was positive in 0.98% of the isolates (2 of 203 isolates). The two *tcpA*-positive strains *V. c-C.chinensis* 717-01, and *V. c-C.chinensis* 805-17 originated from the snail *C. chinensis* (known as the Chinese mystery snail). Amplified *tcpA* gene products were sequenced, and the obtained sequences were deposited in GenBank under the accession numbers MW319058, and MW319059, respectively. Moreover, the *V. c*-*C. chinensis* 717-01 strain also harbored the virulence genes *ace* and *zot*, whose sequences were also determined and deposited in GenBank under the accession numbers MW319061 and MW280619, respectively. In addition, higher incidence of the virulence-associated genes *hapA* (64.8%), *rtxCABD* (56.2%, 41.9%, 65.5%, and 68.0%, respectively), *tlh* (54.2%) and *hlyA* (37.9%) was observed among the 203 isolates. 

**Figure 1:**
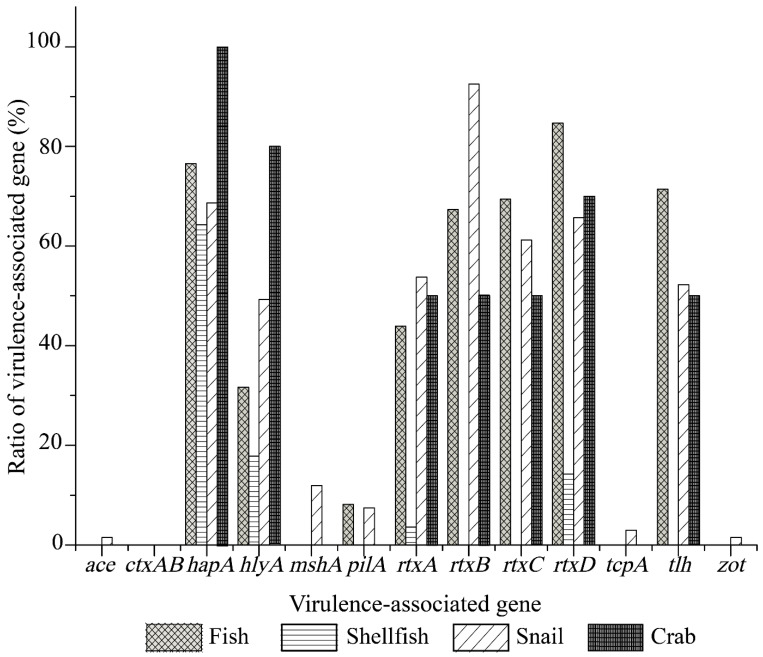
Virulence-associated gene profiles of the *V. cholerae* isolates recovered from the four types of aquatic food animals.

As illustrated in Figure 1, the *V. cholerae* isolates recovered from the four types of aquatic food animals harbored various virulence-associated gene profiles. The *tcpA*, *ace*, *zot*, and *mshA* genes were solely present in the isolates of snail origin with detection rates of 2.9% (2 of 69 isolates), 1.4% (1/69 isolates), 1.4% (1/69 isolates), and 11.6% (8/69 isolates), respectively. Moreover, higher percentages of the *rtxCABD* (89.9–52.2%), *hapA* (66.7%), *tlh* (50.7%), and *hlyA* (47.8%) genes were observed among the isolates of snail origin, whereas the *pilA* gene showed a relatively lower detection rate (7.2%). The isolates recovered from the shellfish samples tested negative for the *pilA*, *rtxBC*, and *tlh* genes, but positive for the *hapA* (62.1%, 18/29 isolates), *hlyA* (17.2%, 5/29 isolates), and *rtxAD* (3.4%, 1/29 isolates; 13.8%, 4/29 isolates) genes. All the isolates of fish origin harbored the *hapA*, *hlyA*, *pilA*, *rtxCABD*, and *tlh* genes with detection frequencies from 87.4% to 8.4%, while these virulence-associated genes except the *pilA* gene were also positive in the isolates from the crab samples (100–50%) (Figure 1).

**Figure 2:**
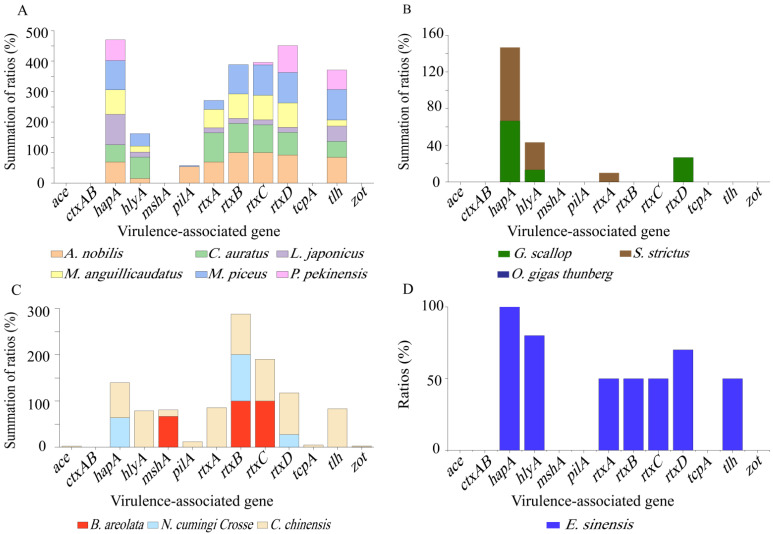
Virulence-associated gene profiles of the *V. cholerae* isolates recovered from the 13 species of aquatic food animals. (**A**–**D**): the species of fish, shellfish, snails, and crab, respectively.

Different virulence-associated gene profiles were also observed among the *V. cholerae* isolates recovered from different species of aquatic food animals (Figure 2A–D). For example, the isolates originating from the snail *C. chinensis* harbored the maximum number (n = 12) of virulence-associated genes tested (Figure 2C), whereas those recovered from the shellfish *O. gigas Thunberg* did not carry any of these genes (Figure 2B). In remarkable contrast to *C. chinensis*, the other two species of snails *B. areolata* (*mshA^+^, rtxB^+^C^+^*) and *N. cumingi Crosse* (*hapA^+^*, *rtxB^+^D^+^*) had a small number (n = 3) of these genes with detection frequencies of 100.0–66.7%, and 100.0–27.3%, respectively (Figure 2C). The same case was observed for the other two species of shellfish *G. scallop* (*hapA^+^, hlyA^+^, rtxD^+^*) and *S. strictus* (*hapA^+^, hlyA^+^, rtxD^+^*) (Figure 2B). Additionally, the incidence of each virulence-associated gene varied depending on the species of aquatic food animals. For example, higher percentages of *hapA* gene (100.0–56.5%) were found in all the species tested (except *O. gigas Thunberg* and *B. areolata*) (Figure 2A–D), whereas the *mshA* gene was solely present in the isolates recovered from *B. areolata* (66.7%) and *C. chinensis* (14.3%) (Figure 2C). Interestingly, the majority of the isolates from *A. nobilis*, *C. auratus*, *M. anguillicaudatus*, *C. chinensis*, and *E. sinensis* harbored *rtxCABD* genes (Figure 2A,C,D), while truncated versions of this gene cluster existed in the other species of aquatic food animals (except *O. gigas Thunberg*) (Figure 2A–C).

The 203 *V. cholerae* isolates had 50 different virulence-associated gene profiles, which highlighted their considerable genetic diversity. Among these, the *hapA^+^hlyA^+^tlh^+^rtxA^+^B^+^C^+^D^+^* gene profile was the most predominant (14.3%, n = 29), followed by the *hapA^+^*(8.4%, n = 17), and *hapA^+^rtxB^+^C^+^D^+^tlh^+^* (7.4%, n = 15). Conversely, seven isolates did not carry any of the virulence-associated genes tested (Table S2).

### 2.3. Antimicrobial Resistance Profiles of the V. cholerae Isolates

**Figure 3:**
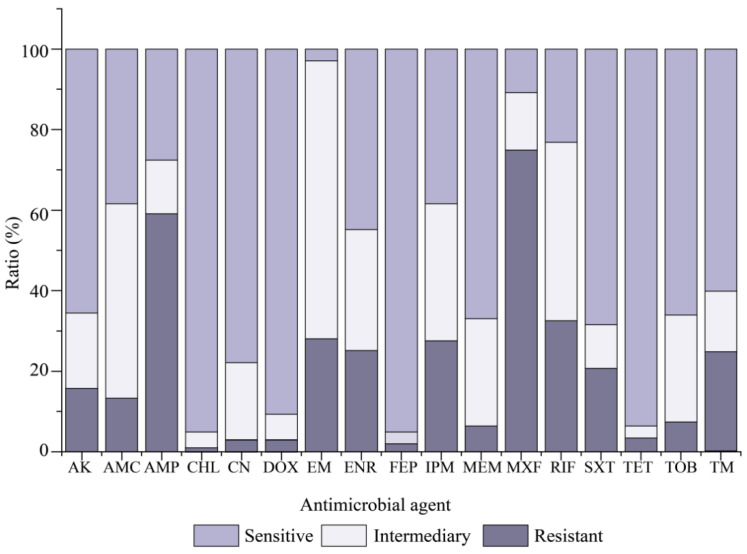
Antimicrobial susceptibility of the 203 *V. cholerae* isolates to 17 antimicrobial agents evaluated in this study. AK, amikacin; AMC, amoxicillin/novobiocin; AMP, ampicillin; CHL, chloramphenicol; CN, gentamicinm; DOX, doxycycline; EM, erythromycin; ENR, enrofloxacin; FEP, cefepime; IPM, imipenem; MEM, meropenem; MXF, moxifloxacin; RIF, rifampin; SXT, sulfamethoxazole- trimethoprim; TET, tetracycline; TOB, tobramycin; and TM, trimethoprim.

Resistance profiles of the 203 *V. cholerae* isolates for 17 commonly used antimicrobial agents were determined, and the results are illustrated in Figure 3. Most isolates were sensitive to CHL (95.1%), FEP (95.1%), TET (93.6%), and DOX (90.6%). In contrast, MXF resistance was the most predominant (74.9%) among the *V. cholerae* isolates, followed by AMP (59.1%) and RIF (32.5%). Additionally, approximately 69.0%, 48.3%, and 44.3% of the isolates also exhibited intermediate susceptibility to EM, AMC and RIF. The resistance trend of the 203 *V. cholerae* isolates was MXF > AMP > RIF > EM > IPM > ENR > TM > SXT > AK > AMC > TOB > MEM > TET > CN > DOX > FEP > CHL (Figure 3).

**Figure 4:**
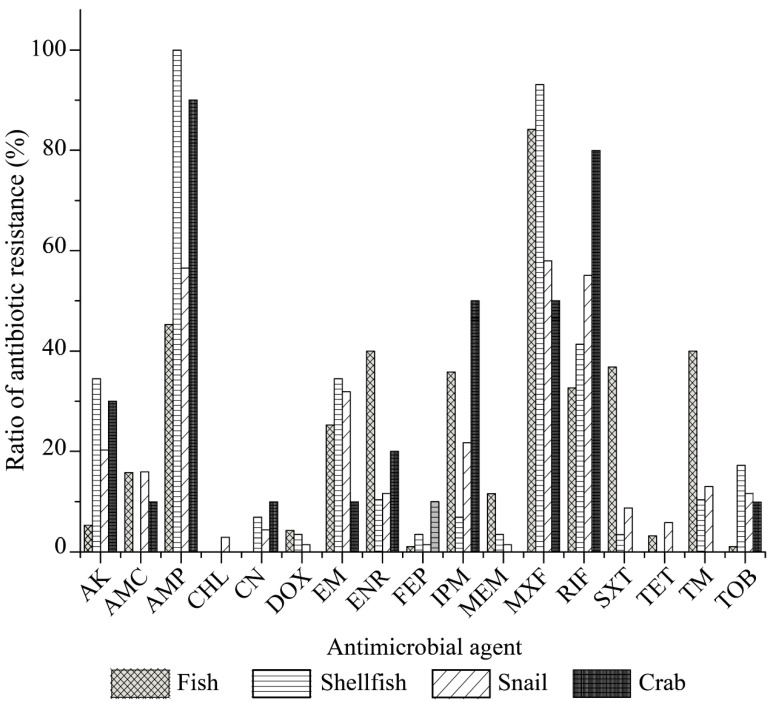
Antimicrobial resistance profiles of the *V. cholerae* isolates in the four types of aquatic food animals.

Our data also revealed distinct antibiotic resistance profiles of the *V. cholerae* isolates originating from the four types of aquatic food animals (Figure 4). All the isolates of the fish origin were sensitive to CHL and CN. Moreover, relatively lower resistance rates were observed to FEP, TOB, TET, DOX and AK (5.3–1.1%). In contrast, MXF resistance was the most prevalent among the isolates of fish origin (84.2%), and higher resistance percentages to AMP, SXT, TM, ENR, IPM, RIF, EM, AMC and MEM (45.3–11.6%) were also observed. Among the isolates of shellfish origin, all were sensitive to AMC, CHL and TET; fewer had resistance to CN, DOX, FEP, IPM, MEM, and SXT (6.9–3.4%); however, most of these isolates displayed resistance to AMP (100%) and MXF (93.1%), and 41.4–10.3% to RIF, AK, EM, TOB, and TM. The *V. cholerae* isolates of snail origin displayed resistance to all 17 antimicrobial drugs examined in this study (58.0–1.45%), while the isolates recovered from the crab samples were resistant to 11 of the 17 drugs (90.0–10.0%), and sensitive to six drugs including CHL, DOX, MEM, SXT, TET, and TM (Figure 4). 

**Figure 5:**
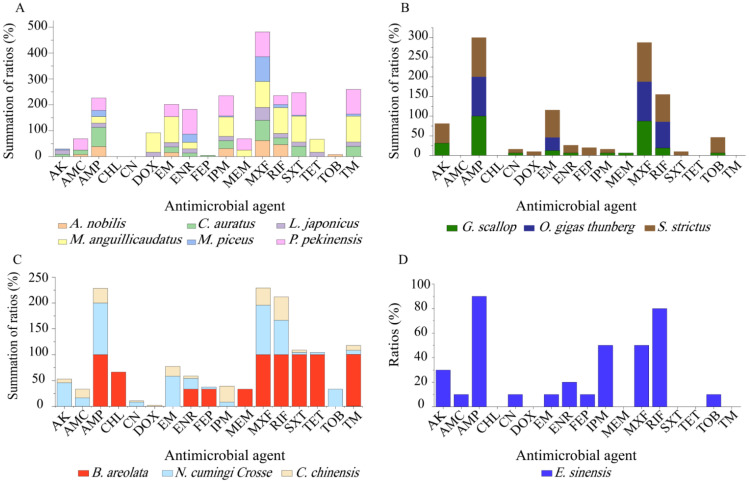
Antimicrobial resistance profiles of the *V. cholerae* isolates in the 13 species of aquatic food animals. (**A**–**D**): the species of fish, shellfish, snails, and crab, respectively.

Although only a small number of *V. cholerae* strains were isolated and tested, different antibiotic resistance profiles were also found among the isolates in the 13 species of aquatic food animals (Figure 5). Resistance to MXF (100–33.3%), AMP (93.1–50%), and RIF (100–12.0%) was very prevalent among the isolates from the 13 species tested (Figure 5A–D). For example, all the isolates recovered from *G. scallop, O. gigas Thunberg*, *S. strictus*, *B. areolate*, and *N. cumingi Crosse* were resistant to AMP (Figure 5B,C), while all from *M. anguillicaudatus, O. gigas thunberg*, *S. strictus*, and *B. areolata* were resistant to MXF (Figure 5A–C). Meanwhile, all the isolates from *M. anguillicaudatus* and *B. areolata* were resistant to RIF (Figure 5A,C). Conversely, resistance to CHL was only found in two isolates (*V. c-B. areolata* 707-19, *V. c-B. areolata* 707-39), which originated from *B. areolata* (Figure 5C). CN resistance was solely observed in six isolates from *G. scallop*, *S. strictus*, *N. cumingi Crosse*, *C. chinensis*, and *E. sinensis* (Figure 5B–D). Additionally, none of the isolates from *M. piceus*, *P. pekinensis*, *G. scallop* or *O. gigas Thunberg* were resistant to DOX, FEP or TET. Resistance to DOX was only found in a few isolates from *M. anguillicaudatus*, *L. japonicas*, *S. strictus*, and *C. chinensis* (Figure 5A–C). FEP resistance was also present in a few isolates from *C. auratus*, *S. strictus*, *B. areolata*, *N. cumingi Crosse*, and *E. sinensis* (Figure 5A–D). Similarly, a small number of the isolates from *M. anguillicaudatus* (n = 2), *L. japonicas* (n = 1), *B. areolata* (n = 3), and *N. cumingi Crosse* (n = 1) had resistance to TET, whereas those from the other 9 species were sensitive to this drug (Figure 5).

### 2.4. MDR Phenotypes of the V. cholerae Isolates

Approximately 58.6% (n = 119) of the *V. cholerae* isolates had MDR phenotypes, which varied depending on different types and species of aquatic food animals. The isolates originating from the crab samples showed the highest occurrence of MDR (90%), followed by those from shellfish (65.5%), fish (55.8%), and snails (55.1%). Moreover, the MDR phenotypes were the most prevalent among the isolates from *M. anguillicaudatus* (100%), *B. areolata* (100%) and *O. gigas Thunberg* (100%), followed by *P. pekinensis* (91.3%), *S. strictus* (90%), and *C. chinensis* (90%). Conversely, the isolates from *M. piceus* (28.0%), *A. nobilis* (23.1%), and *L. japonicas* (16.7%) showed the opposite pattern. 

The multiple antimicrobial resistance index (MARI) values of the 203 *V. cholerae* isolates ranged from 0.000 to 0.590, which indicated varying degrees of exposure to the 17 antimicrobial agents evaluated. The mean MARI values of the isolates originating from the fish, shellfish, crab, and snail samples were 0.214, 0.212, 0.205, and 0.179, respectively. Among the 13 species, the maximum MARI value was derived from the isolates from *B. areolata* (0.450), followed by those from *M. anguillicaudatus* (0.420) and *P. pekinensis* (0.390), whereas the isolates from *M. piceus*, *C. chinensis* and *A. nobilis* had the smaller MARI values of 0.120, 0.120, and 0.128, respectively. Remarkably, one isolate (*V. c-P. pekinensis* 703-62) originating from *P. pekinensis* had the largest MARI value of 0.590, showing resistance to 10 of the 17 drugs tested.

### 2.5. Heavy Metal Tolerance Profiles of the V. cholerae Isolates

Tolerance of the 203 *V. cholerae* isolates to 8 heavy metals was also examined (Table S3). The maximum MICs observed among the isolates were 3200 μg/mL for Pb^2+^; 1600 μg/mL for Cr^3+^, Mn^2+^, Ni^2+^ and Zn^2+^; 800 μg/mL for Cd^2+^; 400 μg/mL for Cu ^2+^; and 100 μg/mL for Hg^2+^, when compared with those of the quality control strain *E. coli* K12. Tolerance to Hg^2+^ was the most prevalent among the isolates (69.0%), followed by tolerance to Pb^2+^ (57.6%), and Zn^2+^ (57.6%), while the opposite pattern was observed for Cr^3+^ (9.4%), Mn^2+^ (9.4%) and Cu^2+^ (10.3%). The tolerance trend of the 203 *V. cholerae* isolates was Hg^2+^ > Pb^2+^=Zn^2+^ > Ni^2+^ > Cd^2+^ > Cu^2+^ > Cr^3+^ = Mn^2+^.

**Figure 6:**
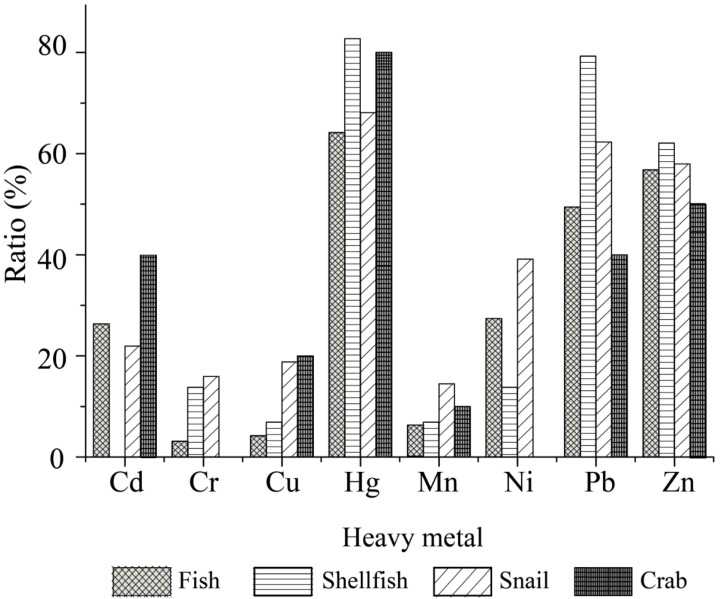
Heavy metal tolerance profiles of the *V. cholerae* isolates recovered from the four types of aquatic food animals.

As shown in Figure 6, the *V. cholerae* isolates originating from the four types of aquatic food animals had different heavy metal tolerance profiles. The majority of the isolates were tolerant to Hg^2+^ (82.8 to 64.2%), and Zn^2+^ (62.1 to 50.0%). Moreover, the percentages of Pb^2+^-tolerant isolates were much higher in shellfish (79.3%) and snails (62.3%) than in fish (49.5%) and crab (40.0%). All the isolates from the crab were susceptible to Cr^3+^ (100%) and Ni^2+^ (100%), whereas those from the snails showed higher tolerance rates to these metals (Cr^3+^, 15.9%; Ni^2+^, 39.1%) than the shellfish (Cr^3+^ and Ni^2+^, 13.8%) and fish (Cr^3+^, 3.2%; Ni^2+^, 27.4%). There were many more Cd^2+^-tolerant isolates from the crab (40.0%) than from fish (26.3%) and snails (21.7%), whereas all the isolates from shellfish were susceptible to this heavy metal. Additionally, relatively lower percentages of Cu^2+^ and Mn^2+^-resistant isolates were found from fish (4.2%, 6.3%), shellfish (6.9%, 6.9%), snails (18.8%, 14.5%), and crab (20.0%, 10.0%), respectively.

**Figure 7:**
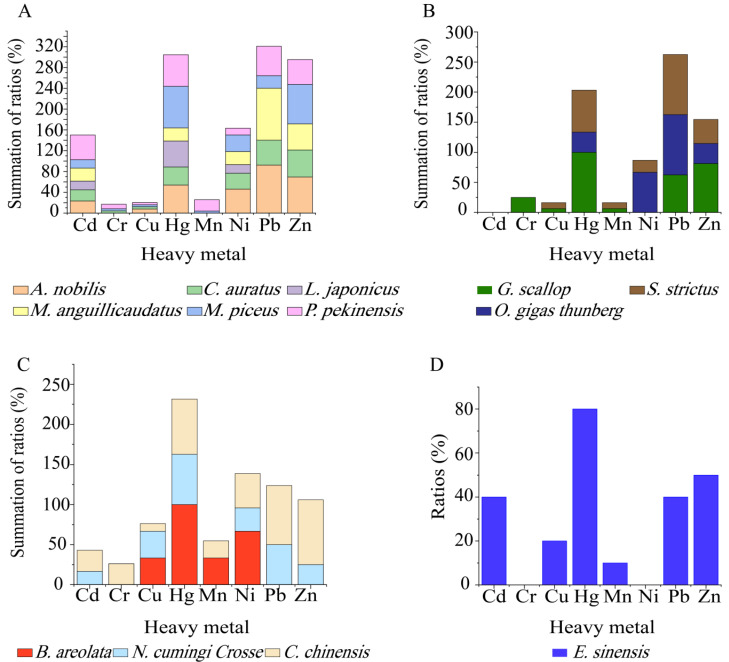
Heavy metal tolerance profiles of the *V. cholerae* isolates recovered from the 13 species of aquatic food animals. (**A**–**D**): the species of fish, shellfish, snails, and crab, respectively.

Different heavy metal tolerance profiles were also found among the *V. cholerae* isolates in the 13 species of aquatic food animals (Figure 7A–D). The isolates from *M. piceus*, *P. pekinensis*, and *C. chinensis* were tolerant to all 8 heavy metals, followed by *C. auratus* (7/8 heavy metals); *A. nobilis*, *G. scallop*, *S. strictus*, *N. cumingi Crosse* and *E. sinensis* (6/8 heavy metals); and *M. anguillicaudatus* (5/8 heavy metals). Moreover, all the isolates from *G. scallop*, and *B. areolata* were resistant to Hg^2+^ (Figure 7B,C), while 80% to 25% of those from the other 11 species were tolerant to this metal (Figure 7A–D). Tolerance to Cr^3+^ was only detected in the isolates from *C. chinensis* (26.2%), *G. scallop* (25%), *P. pekinensis* (8.7%), *C. auratus* (4.4%), and *M. piceus* (4.0%) (Figure 7A–C). Additionally, none of the isolates from *L. japonicus* and *B. areolata* were tolerant to Pb^2+^ and Zn^2+^ (Figure 7A,C), while all the isolates from *G. scallop* and *E. sinensis* were sensitive to Ni^2+^ (Figure 7B,D).

### 2.6. Genetic Diversity of the V. cholerae Isolates

**Figure 8:**
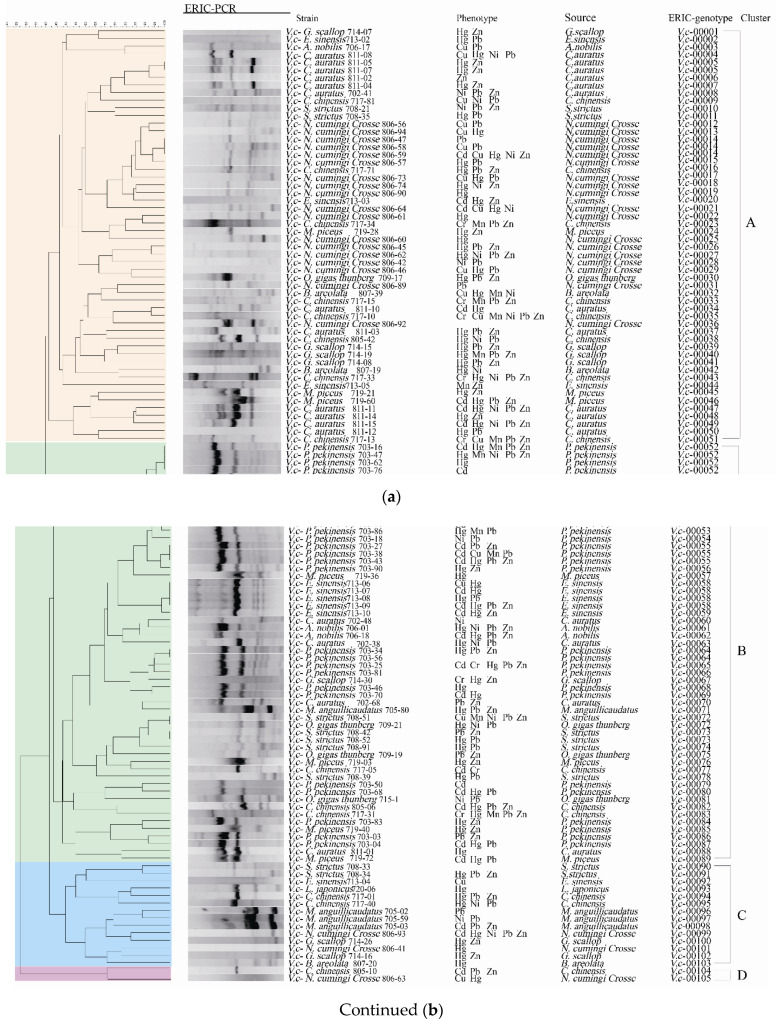
The ERIC-PCR fingerprinting profiles of the MDR *V. cholerae* isolates. (**a**,**b**): half of the profiles.

Enterobacterial repetitive intergenic consensus-PCR (ERIC-PCR) was employed to analyze genetic diversity of the 203 *V. cholerae* isolates originating from the 13 species of aquatic food animals. The obtained genome fingerprinting profiles comprised DNA bands mainly ranging from 100 to 5000 bp (Figure 8). Based on the genome fingerprinting profiles, the 203 isolates were classified into 170 different ERIC-genotypes, approximately 72.9% (n = 148) of which were assigned as singletons. Approximately 40.5% (n = 60), 15.4% (n = 23), 39.9% (n = 59) and 4.1% (n = 6) of these singletons were derived from fish, shellfish, snails, and crab, respectively. The UPGMA algorithm grouped all 170 ERIC-genotypes into nine clusters (Clusters I–IX) at a 43.0% similarity cutoff level (Figure not shown). Approximately 35.5% of the 203 *V. cholerae* isolates were classified into Cluster III, followed by 30.5% and 19.2% into Cluster I and Cluster IV, respectively. The remaining (14.8%) fell into Clusters II, and V to IX (5.9 to 0.5%) (figure not shown). Additionally, most isolates had a Simpson’s diversity index of 0.6458. These results demonstrated a large degree of genetic diversity of the 203 *V. cholera* isolates originating from the 13 species of aquatic food animals.

Approximately 27.1% (n = 55) of the 203 isolates shared 22 ERIC-genotypes. For example, the most predominant ERIC-genotypes *vc*00076 (7.3%) and *vc*00081 (7.3%) were derived from *P. pekinensis* and *E. sinensis*, respectively, suggesting that they were close relatives or had clonal relatedness. Additionally, there were five ERIC-genotypes containing isolates derived from different species of aquatic food animals. For example, three isolates, sharing the ERIC-genotype *Vc*00105, were recovered from *L. japonicus*, *O. gigas Thunberg*, and *S. strictus*. 

### 2.7. Cross-Resistance between Antibiotics and Heavy Metals of the V. cholerae Isolates

To decipher the mechanism underlying cross-selection between antibiotics and heavy metals in *V. cholerae*, we further analyzed the 119 isolates with MDR phenotypes by phylogenetic analysis (Figure 8). The resulting data also revealed considerable genetic diversity of the MDR isolates with a Simpson’s diversity index of 0.6788. These MDR isolates with 105 ERIC-genotypes fell into 4 clusters (Clusters A to D) (Figure 8). Approximately 45.4% of the MDR isolates were grouped into Cluster A (n = 54) with 51 ERIC-genotypes, while Cluster B consisted of 49 MDR isolates with 38 ERIC-genotypes. Smaller numbers of the isolates fell into Cluster C (n = 14) and Cluster D (n = 2). Notably, 81.5% (n = 97) of the 119 MDR isolates were assigned as singletons. Among these, most singletons were derived from fish (n = 41), followed by snails (n = 35), shellfish (n = 16), and crab (n = 5). 

Approximately 96.6% (n = 115) of the MDR *V. cholerae* isolates also had resistance to heavy metals, and the majority of these isolates (81.5%) were tolerant to two or more heavy metals. For example, the isolate *P. pekinensis* 703-62, which displayed resistance to 10 antibiotics (AMC/AMP/EM/ENR/IPM/MEM/MXF/RIF/SXT/TM), was also tolerant to Hg^2+^, while the isolate *C. chinensis* 717-10 tolerant to the maximum number of heavy metals (Cr/Cu/Mn/Ni/Pb/Zn) also showed resistance to AMC/EM/IPM/RIF. Moreover, 11 isolates resistant to nine antibiotics, and they were tolerant to one to three heavy metals. Interestingly, another 11 isolates tolerant to five heavy metals also had resistance to 3–7 antibiotics. These results demonstrated considerable genetic diversity, and cross-resistance relatedness between the MDR properties and heavy metal tolerance of the *V. cholerae* isolates originating from the thirteen species of aquatic food animals examined in this study. 

## 3. Discussion

*V. cholerae* is the leading waterborne pathogen worldwide [2]. Continuous monitoring of *V. cholerae* contamination in aquatic products and identification of risk factors are imperative for food safety control. In this study, we surveyed *V. cholerae* contamination in 36 species of commonly consumed aquatic food animals sold in Shanghai and Fuzhou in July and August of 2019. To our knowledge, the bacterium has not been previously detected in 22 of these species. Our results revealed that *V. cholerae* was present in only 13 species, and was isolated for the first time from *M. anguillicaudatus*, *G. scallop*, *B. areolata*, *C. chinensis*, *N. cumingi Crosse*, and *E. sinensis*. Unexpectedly, the bacterium was also present in a high abundance in the snails (88/427 colonies). To our knowledge, only one study on the detection of *V. cholerae* in snails is available [22]. Serratore et al. reported that 28 batches of edible snails (*Nassarius mutabilis* and *Bolinus brandaris*) in the Adriatic Sea were negative for *E. *coli**, *Vibro vulnificus*, and *V. cholerae* contamination [22]. In this study, our results also provided additional evidence to demonstrate high detection frequencies of *V. cholerae* in fish species such as *A. nobilis*, *C. auratus*, and *P. pekinensis*, consistent with results of our prior research [6,9]. 

The crucial virulence determinants in pathogenic *V. cholerae* are the CT toxin encoded by the temperate filamentous phage CTXΦ [23] and its receptor TCP, used for entry of CTXΦ into the host [24]. ACE and ZOT play key roles in host epithelial disruption during *V. cholerae* infection [25]. Studies have indicated that most *V. cholerae* isolates isolated from the environment do not carry the CT-encoding genes *ctxAB*. Virulence factors associated with the CTX element, such as *zot* and *ace*, were also scarcely found in *V. cholerae* isolates of environmental water origin [26]. For example, Siriphap et al. isolated 16 *V. cholerae* strains from seafood, water, and hand swabs, and found that all the isolates tested negative for *ctxAB, tcpA*, *ace* and *zot* genes [27]. Xu et al. analyzed 400 *V. cholerae* isolates from four species of fish, and found that none of the isolates had the *ctxAB*, *tcpA*, *ace*, or *zot* genes [6]. Fu et al. examined 370 *V. cholerae* isolates from twelve species of aquatic products including nine species of fish, one species of crustacean and two species of shellfish. They found that none of the isolates carried the *ctxAB*, *tcpA, ace,* or *zot* genes [9]. In this study, our results indicated that the *ctxAB* genes were absent from all the *V. cholerae* isolates tested, consistent with results in previous reports. However, approximately 0.98% of the *V. cholerae* isolates harbored the *tcpA* gene. The *tcpA*-positive isolates (*V. c*-*C. chinensis* 717-01 and *V. c*-*C. chinensis* 805-17) originated from the snail *C. chinensis*. The incidence of the *tcpA*-positive isolates was approximately 4.8% in *C. chinensis* (2/42 isolates). One of the *tcpA*-positive isolates (*V. c*-*C. chinensis* 717-01) carried the *ace* and *zot* genes as well. The *ace-ctxB*-F/R primers (Table S4) flanking the *ace-zot-ctxA-ctxB* genes (3.4 kb) on the CTXΦ in *V. cholerae* N16961/ATCC39315 (NCBI accession No. NZ_CP028827, 1,513,053-1,516,480 bp) were designed and synthesized. Genomic DNA of *V. c*-*C. chinensis* 717-01 was used as a template for PCR, and the resulting product (1.7 kb) was determined by DNA sequencing, which corresponds to a defective CTX-Phi lacking the *ctxAB* genes (Figure S1), consistent with the previous research [28,29]. It will be interesting to investigate the function of *ace* and *zot* genes that remains in *V. c*-*C. chinensis* 717-01 in future research. Additionally, the isolates of the *C. chinensis* origin harbored the maximum number (n = 12) of virulence-associated genes tested. These results provide the first experimental evidence for the presence of potentially toxic *V. cholerae* in the snail *C. chinensis*. 

The genes encoding virulence-associated factors contribute to the pathogenicity of *V. cholerae.* Numerous studies have reported their presence in the bacterium of environmental and clinical origins [30,31,32]. In this study, high incidence of virulence-associated genes *hapA* (73.4%), *rtxCABD* (68.0 to 41.9%), *tlh* (54.2%), and *hlyA* (37.9%) was observed among the 203 *V. cholerae* isolates. The *hapA* gene encodes a hemagglutinin protease secreted by *V. cholera* through the type II secretion system that functions in *V. cholerae* interactions with aquatic hosts [7,33]. Studies have reported that most *V. cholerae* strains carry the *hap* gene irrespective of their source (i.e., clinical or environmental) [6,9,34], consistent with our present results. RTX is essential for the cytotoxic activity of *V. cholerae* O1 El Tor strain upon Hep-2 cells in vitro [35,36]. Xu et al. reported high percentages of the *rtxA* (83.0%), *rtxB* (97.0%), *rtxC* (95.8%) and *rtxD* (95.5%) genes in *V. cholera* isolates recovered from four fish species [6]. Fu et al. also found that the majority of 370 *V. cholerae* isolates harbored at least three genes of the *rtxCABD* cluster (81.4%, 24.3%, 80.3%, 80.8%, respectively) [9]. In this study, our results also revealed different detection frequencies of the *rtxA* (41.9%), *rtxB* (65.5%), *rtxC* (56.2%), and *rtxD* (68.0%) genes in the *V. cholera* isolates recovered from the 13 species of aquatic food animals. Interestingly, the majority of the isolates from *A. nobilis*, *C. auratus*, *M. anguillicaudatus*, *C. chinensis*, and *E. sinensis* harbored *rtxCABD* genes, while truncated versions of this gene cluster existed in all the other species (except *O. gigas thunberg*) (Figure 2). The extracellular metalloprotease HlyA can be produced by biotype El Tor of serogroup O1 and most of the non-O1/O139 strains, which show multiple pathogenic activities [37]. The *tlh* gene encodes thermolabile hemolysin with phospholipase and lecithinase activity [38]). Compared with the data reported by Xu et al. and Fu et al. [6,9], the incidence of *tlh* (54.2%) and *hlyA* (37.9%) was relatively lower in the *V. cholerae* isolates tested in this study, and varied depending on the species of aquatic animals. For example, approximately 69.6–80.0% of the *V. cholerae* isolates originated from *C. auratus*, *C. chinensis*, and *E. sinensis* carried the *hlyA* gene, whereas only 0.0–40.7% from the other 10 species. Given that these virulence-related genes are present at different genome loci in *V. cholerae*, various aquatic product matrices and/or aquaculture niches may serve as a possible explanation for their different detection frequencies among the *V. cholerae* isolates. It will be interesting to conduct comparative genomic analysis of the isolates in future research. In total, distinct virulence-associated gene profiles were derived from the *V. cholerae* isolates recovered from the four types and 13 species of aquatic food animals examined in this study. 

The inappropriate use of antibiotics has resulted in considerably increased toxicogenic *V. cholerae* and difficulties in clinical case treatment [39,40]. The aquatic environment is a reservoir of *V. cholerae* and might be an important source of resistant strains. Previous studies have reported antibiotic resistant *V. cholerae* isolates of aquatic product origins [6,9]. In this study, our results indicated that MXF resistance was the most predominant resistance (74.9%) among the *V. cholerae* isolates, more than half of which were also resistant to AMP (59.1%). MXF is a fourth-generation fluoroquinolone antibiotic and frequently detected in surface water environments [41]. Xu et al., and Fu et al. also reported high occurrence (44.5%, and 60.3%) of AMP-resistant *V. cholerae* isolates recovered from aquatic products sampled in the summer of 2017, and 2018 in Shanghai, respectively [6,9]. These data implied that persistent exposure to the antimicrobial drugs occurred in recent years. In addition, in this study, we observed high incidence of intermediate susceptibility to EM (69.0%), AMC (48.3%), and RIF (44.3%), which suggests a potential resistance trend of these drugs. 

Variable antibiotic resistance profiles derived from the *V. cholerae* isolates were observed in different types and species of aquatic food animals. The MARI is often used to determine antibiotic resistance-associated health risks [42]. In this study, the mean MARI values of the isolates originating from the fish, shellfish, crab, and snail samples ranged from 0.214 to 0.179, indicating variable antibiotic exposure levels or contaminated sources of farmed aquatic food animals. Additionally, all the *V. cholerae* isolates originating from *N. cumingi Crosse* were resistant to 14 of 17 antibiotics, followed by those from *S. strictus* (13/17 drugs), *C. chinensis* (12/17 drugs), and *E. sinensis* (12/17 drugs), suggesting heavy exposure of these aquatic food animals to the antibiotics. In total, high detection frequencies of MDR *V. cholerae* isolates analyzed in this study suggested inappropriate usage of antimicrobial agents in the aquaculture, which also imposed potential threat upon human health due to the dissemination of antimicrobial resistance.

Along with rapid industrialization and urbanization, large amounts of heavy metals have been released into the environment [15]. For example, an estimated 2220 tons of mercury are currently emitted to the environment each year by human activity [15]. Previous studies have reported heavy metal residues in waters and sediments of rivers, lakes and oceans, as well as in fish farming environments worldwide, particularly in developing countries [18,19,20]. Heavy metal-tolerant *V. cholerae* isolates in waters and aquatic products have been discovered in prior surveys by our research group [6,9,21,43]. In this study, the results indicated that the *V. cholerae* isolates originating from the 4 types and 13 species of aquatic food animals had different heavy metal tolerance profiles. For example, tolerance to Hg^2+^ was the most predominant among the *V. cholerae* isolates (69.0%), consistent with results of previous research [6,9]. Methylmercury, an organic form of mercury, is a potent neurotoxicant that damages the developing brains of infants and reduces IQ in children [15]. Human exposure to methylmercury occurs primarily through consumption of contaminated fish and marine mammals (WHO, https://www.who.int/, accessed on 31 March 2017) [15]. In this study, high percentages (82.8–64.2%) of Hg^2+^-tolerant *V. cholerae* isolates were observed in all four types of aquatic products. Moreover, all the isolates from *G. scallop*, and *B. areolata* were resistant to Hg^2+^, while 80–25% of those from the other 11 species were tolerant to this metal, implying that variable levels of exposure to Hg pollution occurred in the aquaculture environment. Wang et al. investigated the circumstances of a rice-fish-farming system, and found that Pb, Cd, Hg, As, and Cr were the most damaging heavy metal pollutions in south China [20]. In this study, the isolates recovered from the snails showed the highest tolerance rates to Ni^2+^ (39.1%) and Cr^3+^ (15.9%), while those from the crab were resistant to Cd^2+^ (40.0%). Moreover, all the isolates from *M. piceus*, *P. pekinensis*, and *C. chinensis* were tolerant to 8 heavy metals tested, followed by *C. auratus* (7/8 heavy metals), and *A. nobilis*, *G. scallop*, *S. strictus*, *N. cumingi Crosse,* and *E. sinensis* (6/8 heavy metals). These data suggested that serious heavy metal pollution likely occurred in aquaculture environments, and may originate from urban and industrial wastes, agricultural runoff and sewage, and/or wastewater from treatment plants that finally end up in rivers, estuaries, and oceans. 

Additionally, in this study, comparison of the genome fingerprinting profiles derived from the 119 MDR *V. cholerae* isolates revealed a close relatedness of cross-resistance between antibiotics and heavy metals, suggesting that heavy metal pollution likely coselected for antibiotic resistance, and vice versa. For example, 96.6% of the MDR *V. cholerae* isolates also had resistance to heavy metals, the majority of which (81.5%) were tolerant to two or more heavy metals. Moreover, some MDR isolates displayed tolerance to five or six heavy metals. Due to the pollution of hazardous metals is widespread, worsening, and poorly controlled particularly in low- and middle-income countries. Frequent and persistent heavy metal pollution greatly impacts on the composition of microbial communities. Heavy metals select for metal resistance but can also co-select for resistance to antibiotics [17]. Thus, the results of this study not only provide useful data to fill gaps for policy and research related to risk assessment of *V. cholerae* contamination in aquatic products, but also serve as a reference to better understand the mechanism underlying co-resistance between antibiotics and heavy metals in the other foodborne or waterborne pathogens worldwide.

## 4. Materials and Methods

### 4.1. Sample Collection

A total of 36 species of aquatic food animals were sampled in July and August of 2019 from three large aquatic markets: Jiangyang Aquatic Market (31°21′25.90″ N, 121°26′50.68″ E) and Luchao Port Aquatic Market (30°86′68.58″ N, 121°85′66.88″ E) in Shanghai, and Mawei Aquatic Market (26°00′01.86″ N, 119°47′67.22″ E) in Fuzhou, Fujian Province, China. They included fourteen species of fish, sixteen species of shellfish, four species of snails, one species of crustacean, and one species of crab (Table S1). The 160 samples were collected into sterile sealed bags (Nanjing Maojie Microbial Technology Co., Ltd., Nanjing, China), and immediately transported in ice boxes (700 mm × 440 mm × 390 mm) to our laboratory at Shanghai Ocean University (Shanghai, China) for further analyses. 

### 4.2. Isolation and Identification of V. cholerae

*V. cholerae* was isolated and identified as described previously [6,9,21]. Briefly, 25 g of each fish intestine sample or whole-tissue sample of shellfish, snails, crustaceans, and crab whose shells were removed, was individually rinsed with 225 mL of sterile 1× phosphate buffered saline (PBS, pH 7.4–7.6, Shanghai Sangon Biological Engineering Technology and Services Co., Ltd., Shanghai, China), homogenized, serial diluted ten-fold, and spread onto thiosulfate citrate bile salt sucrose (TCBS; Beijing Land Bridge Technology Co., Ltd., Beijing, China) agar plates and incubated as described previously [6,9,21].

Presumptive *V. cholerae* colonies were identified by arginine dihydrolase and esculin hydrolysis tests [44,45] using double-arginine hydrolase medium and esculin medium (Muwei Biotechnology Co., Ltd., Shanghai, China), respectively. The isolates that tested negative in the two tests, showing deep yellow and brown, respectively, were subjected to the further identification.

The *V. cholerae*-specific gene *lolB* [46] was amplified using the VHMF and VHA-AS5 primers (Table S4) by polymerase chain reaction (PCR) [9]. *V. cholerae* standard strain GIM 1.449 (Guangdong Culture Collection Center, Guangzhou, China) was used as a positive control strain. The bacterial 16S ribosomal RNA (rRNA) gene was amplified using universal bacterial primers 27F and 1492R [6] (Table S4). PCRs were performed and analyzed as described previously [9], and the resulting amplicons were purified and sequenced by Sangon (Shanghai, China). The BLAST of the National Center for Biotechnology Information (NCBI) (https://www.ncbi.nlm.nih.gov/, accessed on 1 July 2019) was used to analyze DNA sequences. 

### 4.3. Detection of Virulence and Virulence-Associated Genes

*V. cholerae* isolates were individually incubated in 5 mL of tryptic soy broth (TSB) (Land Bridge, Beijing, China) (pH 8.5, 3.0% NaCl) at 37°C for 16–18 h. Genomic DNA of the isolates was prepared using the TaKaRa MiniBEST Bacterial Genomic DNA Extraction Kit Ver.3.0 (TaKaRa Biomedical Technology Co., Ltd., Beijing, China) following the manufacturer’s instructions. Extracted DNA samples were analyzed as described previously [47]. 

The virulence and virulence-associated genes *ctxAB*, *tcpA*, *ace*, *zot*, *rtxCABD*, *hapA*, *hlyA*, *tlh*, *mshA*, and *pilA* were detected by PCR assay using the oligonucleotide primers [6,48,49,50,51,52] listed in Table S4. PCRs were performed as described previously [6]. Genomic DNA of *V. cholerae* ATCC 14035 (*ctxAB*^+^, *tcpA*^+^, *ace*^+^, and *zot*^+^) and GIM 1.449 (*ctxAB*^−^, *rtxCABD*^+^, *hlyA*^+^, *tlh*^+^, and *hapA*^+^) were used as positive controls as described previously [6,47]. Amplicons were confirmed by DNA sequencing at Sangon (Shanghai, China). All oligonucleotide primers (Table S4) were synthesized by Sangon (Shanghai, China).

### 4.4. Antibiotic Susceptibility Assay

*V. cholerae* isolates were measured for in vitro susceptibility to 17 antimicrobial agents (Oxoid, UK) as described previously [6,9,21,53]. The 17 drugs included amikacin (AK, 30 μg), amoxicillin/novobiocin (AMC, 30 μg), AMP (10 μg), chloramphenicol (CHL, 30 μg), gentamicin (CN, 10 μg), doxycycline (DOX, 30 μg), erythromycin (EM, 15 μg), enrofloxacin (ENR, 5 μg), cefepime (FEP, 30 μg), imipenem (IPM, 10 μg), meropenem (MEM, 10 μg), moxifloxacin (MXF, 5 μg), RIF (5 μg), SXT (25 μg), tetracycline (TET, 30 μg), tobramycin (TOB, 10 μg), and trimethoprim (TM, 5 μg) (Oxoid, UK). *Escherichia coli* ATCC 25922 (Institute of Industrial Microbiology, Shanghai, China) was used as a quality control strain in the antibiotic susceptibility assay [6,9,21,53].

### 4.5. Heavy Metal Tolerance Assay

A heavy metal tolerance assay was carried out according to a method described previously [6,9,53]. Eight heavy metals CdCl_2_, CrCl_3_, CuCl_2_, NiCl_2_, HgCl_2_, MnCl_2_, PbCl_2_, and ZnCl_2_ were purchased from Analytical Reagent, Sinopharm Chemical Reagent Co., Ltd., Shanghai, China). A broth dilution test (microdilution) was used to measure the minimal inhibitory concentration (MIC) of heavy metals (3200 to 3.125 μg/mL) against the isolates in vitro. *E. coli* K12 (Institute of Industrial Microbiology, Shanghai, China) was used as a quality control strain [6,9,53].

### 4.6. ERIC-PCR Assay

The ERIC-PCR assay was performed with the ERIC1R and ERIC2 primers (Table S4) according to the method described previously [6,9]. Six microliters of each amplicon were analyzed by agarose gel electrophoresis at 100 V for approximately 45 min on 1.0% agarose gels. The ERIC-PCR products were visualized and recorded as described previously [6,9]. The ERIC-PCR fingerprinting patterns were analyzed using BioNumericsc 7.6 software [54], based on unweighted pair groups with arithmetic averages (UPGMA) as described previously [6,9]. 

### 4.7. Statistical Analysis

Data analysis was performed using SPSS statistical analysis software version 17.0 (SPSS Inc., Chicago, IL, USA). The MARI of the isolates was defined as a/b, where a represents the number of antibiotics to which the isolate was resistant, and b represents the number of antibiotics to which the isolate was subjected, and calculated as described previously [6,9,55].

## 5. Conclusions

In the present study, 36 species of aquatic food animals were sampled in July and August of 2019 in Shanghai and Fujian, China. *V. cholerae* has not been previously detected in 22 species of the aquatic animals analyzed here. The bacterium was present in 13 species including *A. nobilis*, *C. auratus*, *L. japonicas*, *M. anguillicaudatus*, *M. piceus*, *P. pekinensis*, *G. scallop*, *O. gigas Thunberg*, *S. strictus*, *B. areolata*, *C. chinensis*, *N. cumingi Crosse*, and *E. sinensis*. *V. cholerae* occurred in six of these species where it wasn’t ever detected before. 

None of the 203 isolates harbored the *ctxAB* gene (0.0%), but they might be potentially harmful because some of them (only a minority) harbored the TCP island, which makes them CTX-sensitive. The *tcpA*-positive isolates (*V. c*-*C. chinensis* 717-01 and *V. c*-*C. chinensis* 805-17) originated from the snail *C. chinensis*. One of the *tcpA*-positive isolates (*V. c*- *C. chinensis* 717-01) had *ace* and *zot* genes as well. Moreover, the isolates of *C. chinensis* origin harbored the maximum number (n = 12) of the virulence-associated genes tested. This study is the first to provide experimental evidence for the presence of potentially toxic *V. cholerae* in *C. chinensis*. Additionally, high incidence of virulence-associated genes was observed among the 203 *V. cholerae* isolates, including *hapA* (73.4%), *rtxCABD* (68.0–41.9%), *tlh* (54.2%), and *hlyA* (37.9%).

High percentages of resistance to MXF (74.9%), AMP (59.11%), and RIF (32.51%) were found among the *V. cholerae* isolates. Approximately 58.6% of the isolates displayed MDR phenotypes. In contrast, most isolates were sensitive to CHL (95.1%), FEP (95.1%), TET (93.6%), and DOX (90.6%). Meanwhile, 67.0% of the *V. cholerae* isolates were tolerant to the heavy metal Hg^2+^, followed by Pb^2+^ (57.6%) and Zn^2+^ (57.6%). Distinct virulence and cross-resistance profiles were discovered among the *V. cholerae* isolates originating from the four types and 13 species of aquatic food animals, suggesting varied exposure levels to the 17 antimicrobial agents and 8 heavy metals evaluated in this study. 

The 203 *V. cholerae* isolates had 170 ERIC-genotypes with considerable genomic variation. Additionally, 96.6% of the 119 MDR isolates tolerated one or more heavy metals. In future research, the mechanism underlying the cross-selection between heavy metals and antibiotics should be further deciphered in *V. cholerae*, a leading waterborne pathogen worldwide.

## Data Availability

The raw sequences generated for this study are available from the NCBI GenBank under the accession numbers MW319058, MW319059, MW319061, and MW280619.

## Supplementary Materials

The following are available online at https://www.mdpi.com/article/10.3390/antibiotics10040412/s1, Table S1. The 36 species of aquatic food animals and the recovered *V. cholerae* isolates, Table S2. Representative virulence genotypes of 203 *V. cholerae* isolates, Table S3. Tolerance of the 203 *V. cholerae* isolates to eight heavy metals, Table S4. The oligonucleotide primers used in this study. Figure S1. Gene organization of the defective CTX-Phi in *V. cholerae*-*C. chinensis* 717-01 strain.
