# Supplementary material for: First Experimental Evidence for the Presence of Potentially Toxic Vibrio cholerae in Snails, and Virulence, Cross-Resistance and Genetic Diversity of the Bacterium in 36 Species of Aquatic Food Animals
Source: Antibiotics (Basel). 2021 Apr 9;10(4):412. doi: 10.3390/antibiotics10040412 (PMC8069825; doi:10.3390/antibiotics10040412)
Supplement: Supplementary file 1 [file antibiotics-10-00412-s001.pdf]

antibiotics

MDPI

*Article*

# First Experimental Evidence for the Presence of Potentially Toxic *Vibrio cholerae* in Snails, and Virulence, Cross-Resistance and Genetic Diversity of the Bacterium in 36 Species of Aquatic Food Animals

**Dailing Chen [1,†], Xiangyi Li [1,†], Ling Ni [1], Dingxiang Xu [1], Yingwei Xu [1], Yong Ding [1], Lu Xie [2,*] and Lanming Chen [1,*]**

[1] Key Laboratory of Quality and Safety Risk Assessment for Aquatic Products on Storage and Preservation (Shanghai), Ministry of Agriculture and Rural Affairs of the People's Republic of China, College of Food Science and Technology, Shanghai Ocean University, Shanghai 201306, China; 180310748@st.shou.edu.cn (D.C.); Lixy376@mail2.sysu.edu.cn (X.L.); lni@shou.edu.cn (L.N.); 190300759@st.shou.edu.cn (D.X.); 190300760@st.shou.edu.cn (Y.X.); yding@shou.edu.cn (Y.D.)
[2] Shanghai Center for Bioinformation Technology, Shanghai 201203, China
* Correspondence: luxiex2017@outlook.com (L.X.); lmchen@shou.edu.cn (L.C.)
† These authors have contributed equally to this work.

**Table S1.** The 36 species of aquatic food animals and the recovered *V. cholerae* isolates.

| Type | Species | Common name | No. of isolates |
|---|---|---|---|
| Fish | *Aristichthys nobilis* | Silver carp | 13 |
| | *Blotchy rock Cod* | Blotchy rock cod | 0 |
| | *Carassius auratus* | Crucian carp | 23 |
| | *Ditrema temmincki Bleeker* | -[1] | 0 |
| | *Hemiculter leucisculus* | White semiknife carp | 0 |
| | *Lateolabrax japonicus* | Weever (striped bass) | 6 |
| | *Misgurnus anguillicaudatus* | - | 4 |
| | *Monopterus albus* | Ricefield eel | 0 |
| | *Mylopharyngodon piceus* | Black carp | 25 |
| | *Nibea albiflora* | Nibea albiflora | 0 |
| | *Ophiocephalus argus Cantor* | - | 0 |
| | *Parabramis pekinensis* | Bream | 23 |
| | *Pelteobagrus fulvidraco* | Yellow forehead fish | 0 |
| | *Plectorhynchus cinctus* | - | 0 |
| Shellfish | *Anodonta woodiana* | River mussel | 0 |
| | *Antigona lamellaris* | Diagonal clam | 0 |
| | *Atrina pectinata* | - | 0 |
| | *Golden scallop* | Golden scallop | 16 |
| | *Haliotis rubra* | Abalone | 0 |
| | *Mactra antiquata* | Sea mussel | 0 |
| | *Mactra veneriformis* | White clam | 0 |
| | *Musculus senhousei* | Muscle clam | 0 |
| | *Mytilus edulis* | Mussel | 0 |
| | *Ostrea gigas thunberg* | Oyster | 3 |

| | | | |
|---|---|---|---|
| | *Pseudocardium sachalinense* | Sakhalin maca | 0 |
| | *Saxidomus purpuratus* | - | 0 |
| | *Scapharca subcrenata* | Cockles | 0 |
| | *Sinonovacula constricta* | Razor fish | 0 |
| | *Solen strictus* | Razor clam | 10 |
| | *Tegillarca granosa* | Blood clam | 0 |
| Snail | *Babylonia areolata* | - | 3 |
| | *Babylonia lutosa* | - | 0 |
| | *Cipangopaludina chinensis* | Chinese mystery snail | 42 |
| | *Neptunea cumingi Crosse* | - | 24 |
| Shrimp | *Procambarus clarkii* | Crayfish | 0 |
| Crab | *Eriocheir sinensis* | Chinese mitten crab | 10 |
| Total | 36 | 36 | 203 |

[1]: not available.

**Table S2.** Representative virulence-associated gene profiles of the 203 *V. cholerae* isolates.

| No. of gene | Genotype | No. of isolate |
|---|---|---|
| 0 | ~ | 7 |
| 1 | *hapA*$^+$ | 17 |
| | *hlyA*$^+$ | 1 |
| | *rtxB*$^+$ | 2 |
| | *rtxD*$^+$ | 6 |
| 2 | *hapA*$^+$/*hlyA*$^+$ | 7 |
| | *hapA*$^+$/ *rtxA*$^+$ | 1 |
| | *hapA*$^+$/ *rtxB*$^+$ | 14 |
| | *hapA*$^+$/ *rtxD*$^+$ | 6 |
| | *hapA*$^+$/ *tlh*$^+$ | 5 |
| | *rtxB*$^+$/*rtxC*$^+$ | 1 |
| | *rtxB*$^+$/ *rtxD*$^+$ | 6 |
| | *rtxD*$^+$/*tlh* | 3 |
| 3 | *hapA*$^+$/ *rtxB*$^+$/*rtxC*$^+$ | 1 |
| | *hapA*$^+$/*hlyA*$^+$/ *rtxD*$^+$ | 2 |
| | *hapA*$^+$/ *rtxD*$^+$/*tlh* $^+$ | 9 |
| | *rtxC*$^+$/*rtxD*$^+$/ *tlh*$^+$ | 1 |
| | *hlyA*$^+$/*mshA*$^+$/ *tlh*$^+$ | 1 |
| | *mshA*$^+$/ *rtxB*$^+$/*rtxC*$^+$ | 2 |
| | *rtxA*$^+$/*rtxB*$^+$/*rtxC*$^+$ | 1 |
| 4 | *hapA*$^+$/ *hlyA*$^+$/*rtxA*$^+$/ *rtxC*$^+$ | 1 |
| | *hapA*$^+$/ *rtxB*$^+$/*rtxC*$^+$/*rtxD*$^+$ | 3 |
| | *hapA*$^+$/*rtxC*$^+$/*rtxD*$^+$/ *tlh* $^+$ | 2 |
| | *hlyA*$^+$/ *rtxB*$^+$/*rtxC*$^+$/*rtxD*$^+$ | 1 |
| | *rtxA*$^+$/*rtxB*$^+$/*rtxC*$^+$/*rtxD*$^+$ | 1 |
| | *rtxB*$^+$/*rtxC*$^+$/*rtxD*$^+$/ *tlh* $^+$ | 1 |
| 5 | *hapA*$^+$/*hlyA*$^+$/ *rtxA*$^+$/*rtxB*$^+$/*rtxC*$^+$ | 1 |
| | *hapA*$^+$/ *rtxA*$^+$/*rtxB*$^+$/*rtxC*$^+$/*rtxD*$^+$ | 2 |
| | *hapA*$^+$/ *rtxA*$^+$/*rtxB*$^+$/*rtxC*$^+$/*tlh*$^+$ | 1 |
| | *hapA*$^+$/ *rtxB*$^+$/*rtxC*$^+$/*rtxD*$^+$/*tlh*$^+$ | 15 |
| | *hlyA*$^+$/*rtxA*$^+$/*rtxB*$^+$/*rtxC*$^+$/*rtxD*$^+$ | 5 |

| | | |
|---|---|---|
| | *hlyA⁺/rtxA⁺/rtxB⁺/rtxC⁺/ tlh⁺* | 1 |
| | *rtxA⁺/rtxB⁺/rtxC⁺/rtxD⁺/ tlh⁺* | 4 |
| 6 | *hapA⁺/hlyA⁺/ rtxA⁺/rtxB⁺/rtxC⁺/rtxD⁺* | 3 |
| | *hapA⁺/hlyA⁺/tlh⁺/rtxA⁺/rtxB⁺/rtxC⁺* | 1 |
| | *hapA⁺/hlyA⁺/tlh⁺/rtxA⁺/rtxB⁺/rtxD⁺* | 2 |
| | *hapA⁺/hlyA⁺/tlh⁺/rtxA⁺/rtxC⁺/rtxD⁺* | 1 |
| | *hapA⁺/hlyA⁺/tlh⁺/rtxB⁺/rtxC⁺/rtxD⁺* | 5 |
| | *hapA⁺/tlh⁺/rtxA⁺/rtxB⁺/rtxC⁺/rtxD⁺* | 6 |
| | *hlyA⁺/tlh⁺/rtxA⁺/rtxB⁺/rtxC⁺/rtxD⁺* | 6 |
| | *pilA⁺/tlh⁺/rtxA⁺/rtxB⁺/rtxC⁺/rtxD⁺* | 2 |
| 7 | *hapA⁺/hlyA⁺/tlh⁺/rtxA⁺/rtxB⁺/rtxC⁺/rtxD⁺* | 29 |
| | *hapA⁺/hlyA⁺/mshA⁺/rtxA⁺/rtxB⁺/rtxC⁺/rtxD⁺* | 1 |
| | *hapA⁺/pilA⁺/tlh⁺/rtxA⁺/rtxB⁺/rtxC⁺/rtxD⁺* | 6 |
| | *hlyA⁺/pilA⁺/tlh⁺/rtxA⁺/rtxB⁺/rtxC⁺/rtxD⁺* | 1 |
| | *hlyA⁺/pilA⁺/mshA⁺/rtxA⁺/rtxB⁺/rtxC⁺/rtxD⁺* | 1 |
| 8 | *hapA⁺/hlyA⁺/mshA⁺/tlh⁺/rtxA⁺/rtxB⁺/rtxC⁺/rtxD⁺* | 3 |
| | *hapA⁺/hlyA⁺/pilA⁺/tlh⁺/rtxA⁺/rtxB⁺/rtxC⁺/rtxD* | 3 |
| | *hapA⁺/hlyA⁺/tcpA⁺/tlh⁺/rtxA⁺/rtxB⁺/rtxC⁺/rtxD* | 1 |
| 9 | *ace⁺/hapA⁺/ rtxA⁺/rtxB⁺/rtxC⁺/rtxD⁺/ tlh ⁺/zot⁺/tcpA⁺* | 1 |

**Table S3.** Tolerance of the 203 *V. cholerae* isolates to the eight heavy metals.

| Heavy metal | Number of isolates with a maximum observed MIC (μg/mL) | | | | | | | | | | | Resistance | |
|---|---|---|---|---|---|---|---|---|---|---|---|---|---|
| | 3.125 | 6.25 | 12.5 | 25 | 50 | 100 | 200 | 400 | 800 | 1600 | 3200 | No. | % |
| $Cd^{2+}$ | | | | 1 | 28 | 67 | a[1] 63 | 27 | 17 | | | 44 | 21.7 |
| $Cr^{3+}$ | | | | | 1 | 3 | 4 | 33 | a 143 | 19 | | 19 | 9.4 |
| $Cu^{2+}$ | | | | 6 | 47 | 90 | a 39 | 21 | | | | 21 | 10.3 |
| $Hg^{2+}$ | 5 | a 58 | 83 | 40 | 9 | 8 | | | | | | 140 | 69.0 |
| $Mn^{2+}$ | | | | | 1 | 7 | 11 | 51 | a 114 | 19 | | 19 | 9.4 |
| $Ni^{2+}$ | | | | | 13 | 56 | a 77 | 54 | 2 | 1 | | 57 | 28.1 |
| $Pb^{2+}$ | | | | | | 1 | 7 | 16 | a 62 | 110 | 7 | 117 | 57.6 |
| $Zn^{2+}$ | | | | | 2 | 7 | 22 | a 55 | 95 | 22 | | 117 | 57.6 |

[1]: a, minimal inhibition concentration of the standard quality control strain *E. coli* K12

**Table S4.** Oligonucleotide primers used in this study.

| Primer | Sequence (5'-3') | Predicted amplicon size (bp) | References |
|---|---|---|---|
| VHMF | TGGGAGCAGCGTCCATTGTG | 516 | [44] |
| VHA-AS5 | CAATCACACCAAGTCACTC | | |
| *ctxAB*-F | TGAAATAAAGCAGTCAGGTG | 778 | [46] |
| *ctxAB*-R | GGTATTCTGCACACAAATCAG | | |
| *tcpA*-F | ATGCAATTATTAAAACAGCTTTTTAAG | 675 | [47] |
| *tcpA*-R | TTAGCTGTTACCAAATGCAACAG | | |
| *ace*-F | TAAGGATGTGCTTATGATGGACACCC | 316 | [48] |
| *ace*-R | CGTGATGAATAAAGATACTCATAGG | | |
| *zot*-F | TCGCTTAACGATGGCGCGTTTT | 947 | [49] |
| *zot*-R | AACCCCGTTTCACTTCTACCCA | | |
| *rtxA*-F | GGGATACAATGCCCTCTGGCA | 977 | [50] |
| *rtxA*-R | TGGGTTGGCGGTTGGATTTTAC | | |
| *rtxB*-F | ATTCATTTTTATTTAAGTGTCATCA | 400 | [6] |
| *rtxB*-R | TTTCGCTCAGCACTCTTT | | |
| *rtxC*-F | ATGTCTATTACACATCAACCTGCAA | 437 | [6] |
| *rtxC*-R | CGGATACAGCGGTCATTT | | |
| *rtxD*-F | ATCATGAAGCGTTTCTTTGGTCAAA | 334 | [6] |
| *rtxD*-R | CGCCCAAGGTATCAAGAGTCAG | | |
| *tlh*-F | TGGGAGTGGGCAAAGAAT | 274 | [6] |
| *tlh*-R | AAAGGCTATCGCCAAACG | | |
| *hlyA-F* | CCAAGTGGTGAAGCGGCGGAC | 393 | [47] |
| *hlyA-R* | TTCGCTGTTTGCCGGTGCCG | | |
| *hapA-F* | CGTTAGTGCCCATGAGGTC | 207 | [6] |
| *hapA-R* | CGTGACGGCTGATCGAAAT | | |
| *pilA-F* | GCGATTGCAATTCCTCAA | 227 | [6] |
| *pilA-R* | CCTAATGCACCTGATGCT | | |
| *mshA-F* | CGCTAGATACTTCGAGTGAG | 189 | [6] |
| *mshA-R* | TACCACAAGCAGTTCCAG | | |
| 27F | GAGAGTTTGATCCTGGCTCAG | ～1500 | [6] |
| 1492R | TACGGCTACCTTGTTACGAC | | |
| ERIC1R | ATGTAAGCTCCTGGGGATTCAC | | [6] |
| ERIC2 | AAGTAAGTGACTGGGGTGAGCG | | |
| *ace-ctxB*-F | TTTACGTGGCTTGTGATC | 3125 | This study |
| *ace-ctxB*-R | GCTGTCGCAGTATAGAGGT | | |

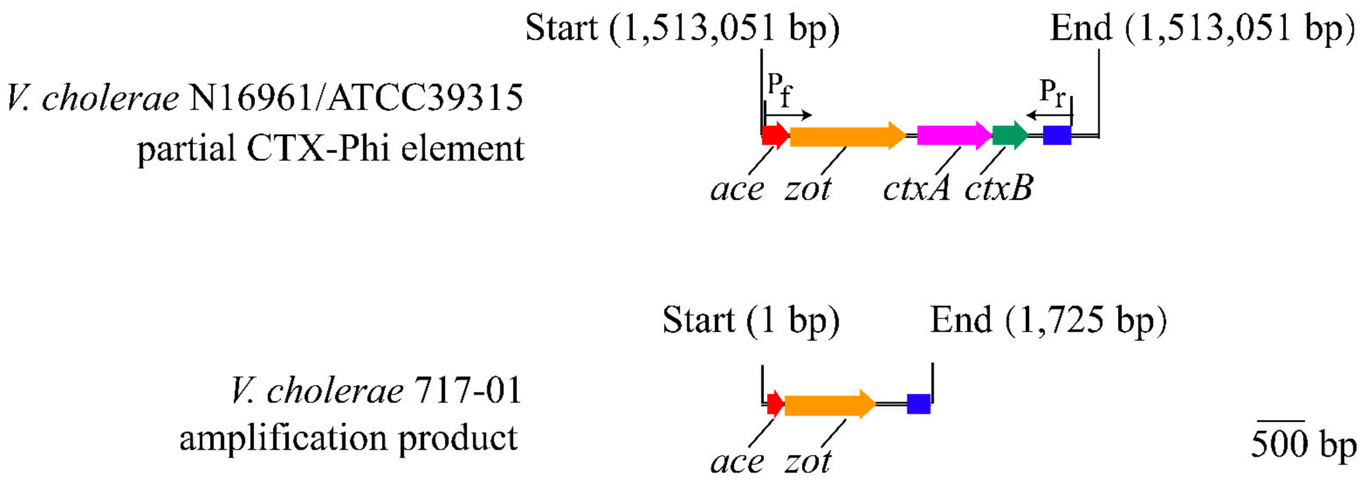


**Figure S1.** Gene organization of the defective CTX-Phi in *V. cholerae-C. chinensis* 717-01 strain.
